# Age at menarche and epithelial ovarian cancer risk: A meta‐analysis and Mendelian randomization study

**DOI:** 10.1002/cam4.2315

**Published:** 2019-05-30

**Authors:** Huijun Yang, Hongji Dai, Lian Li, Xin Wang, Peishan Wang, Fengju Song, Ben Zhang, Kexin Chen

**Affiliations:** ^1^ Tianjin Key Laboratory of Cancer Prevention and Therapy, Department of Epidemiology and Biostatistics, National Clinical Research Center for Cancer, Tianjin Medical University Cancer Institute and Hospital Tianjin Medical University Tianjin China; ^2^ Department of Epidemiology and Biostatistics, First Affiliated Hospital and Southwest School of Medicine Army Medical University Chongqing China

**Keywords:** menarche, Mendelian randomization analysis, meta‐analysis, ovarian cancer

## Abstract

Age at menarche (AAM) was found to be associated with ovarian cancer risk in previous observational studies. However, the causality of this association remains unclear. Here, after systematic meta‐analyses, we performed two‐sample Mendelian randomization (MR) analyses to evaluate the causal effect of AAM in epithelial ovarian cancer (EOC) etiology. We performed meta‐analyses including 11 410 cases and 1 163 117 noncases to quantitatively evaluate the association between AAM and ovarian cancer risk. In MR analyses, we used 25 single nucleotide polymorphisms (SNPs) associated with AAM for Chinese and 390 SNPs for Europeans as instrumental variables. MR estimates were calculated using inverse‐variance weighted methods from 1044 cases and 1172 controls in a Chinese genome‐wide association study and validated by the Ovarian Cancer Association Consortium and Consortium of Investigators of Modifiers of BRCA1/2 studies with 29 396 cases and 68 502 controls of European ancestry. In meta‐analyses, we observed an inverse association (odds ratio [OR] = 0.96, 95% confidence interval [CI] = 0.93 to 1.00, *P* = 0.036) between per year older AAM and ovarian cancer risk in case–control studies, but no association was observed in cohort studies. In MR analyses, the OR of EOC risk per year increase in AAM was 0.81 (95% CI = 0.67 to 0.97, *P* = 0.026) in Chinese and 0.94 (95% CI = 0.90 to 0.98, *P* = 0.003) in Europeans, respectively. Our study supports a causal association between AAM and EOC risk.

## 1 INTRODUCTION

Ovarian cancer accounts for approximately 295 400 new cases and 184 800 deaths worldwide in 2018.^1^ Epithelial ovarian cancer (EOC) accounting for about 90% in all clinical cases develops from the cells lining the surface or epithelium of the ovaries.^2^ As the major histological subtype of EOC, serous ovarian cancer (SOC) accounting for more than 70% in all EOC cases originates from serous cells.^2^ Hormonal and reproductive factors may be associated with ovarian cancer risk.^3^ Age at menarche (AAM), as an observable factor reflecting pubertal hormonal levels, is influenced by genetic, epigenetic, and environmental factors.^4^ Although previous observational studies suggested AAM was associated with risk of ovarian cancer,^5^, ^6^, ^7^, ^8^ the association for per year effect of AAM had not been studied, and traditional observational studies could suffer from methodological flaws and confounding bias.

Mendelian randomization (MR) study, using genetic factors as instrumental variables for the exposure, can estimate the causal association between an exposure and an associated outcome.^9^, ^10^ It takes advantage over the potential to reduce confounder bias and eliminate reverse causality.^11^ Compared to one‐sample MR approach, the two‐sample MR approach can be efficient and powerful for obtaining "gene‐risk factor" and "gene‐outcome" associations from two independent samples in the same population.^12^ AAM has a substantial heritability.^13^ Robust genetic variants (eg, single nucleotide polymorphisms [SNPs]) have been identified by AAM genome‐wide association studies (GWAS) in both European and Asian populations.^14^, ^15^, ^16^, ^17^, ^18^, ^19^, ^20^, ^21^, ^22^, ^23^, ^24^ The genes identified by AAM GWAS were reported to be related to the Hypothalamus‐Pituitary‐Gonadal (HPG) axis, which acts on the secretion and regulation of hormones.^25^ Thus, it is possible to use the AAM‐associated genetic variants as instrumental variables to examine the effect of AAM in ovarian cancer etiology.^26^ A previous MR analysis in Europeans 15 indicated a possible association between AAM and risk of ovarian cancer, but the results might be biased by pleiotropic effects. Therefore, it is worthy to evaluate the causal effect of AAM in ovarian cancer etiology in other populations, and a larger study including more ovarian cancers in Europeans is also warranted.

Here, we performed meta‐analyses pooling 26 studies including more than 1.1 million participants to assess the association between per year older AAM and ovarian cancer risk. In order to test the relationship between AAM and EOC risk, we performed two‐sample MR analyses using our previous EOC GWAS data in Chinese and validated the results in women of European ancestry.

## 2 METHODS

### 2.1 Meta‐analysis of observational studies

**Figure 1:**
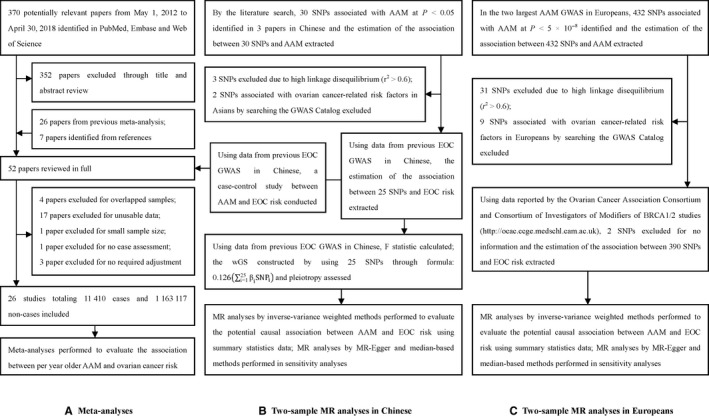
Overview of the study design (A) meta‐analyses (B) two‐sample MR analyses in Chinese (C) Two‐sample MR analyses in Europeans. This figure details the process of meta‐analyses and MR analyses in this study. A, The literature screening in meta‐analyses includes the collection and selection of potentially relevant papers. B, The process of two‐sample MR study in Chinese includes the selection of instrumental variables, calculation of *F* statistic, construction of wGS, MR association analyses, and sensitivity analyses. C, The process of two‐sample MR study in Europeans includes the selection of instrumental variables, MR association analyses, and sensitivity analyses. Abbreviations: AAM, age at menarche; EOC, epithelial ovarian cancer; GWAS, genome‐wide association study; MR, Mendelian randomization; SNPs, single nucleotide polymorphisms; wGS, weighted genetic score

This meta‐analysis was conducted according to the Preferred Reporting Items for Systematic reviews and Meta‐Analyses guidelines.^27^ We comprehensively searched PubMed, Embase, and Web of Science from 1 May 2012 to 30 April 2018 for epidemiological studies investigating the association between AAM and ovarian cancer risk. The key terms searched were ("menarche") AND ("ovarian" OR "ovary") AND ("cancer" OR "neoplasm" or "carcinoma" OR "malignancy" OR "tumor"). 1 May 2012 was selected as the start date because we included all the referenced studies in a previous meta‐analysis conducted before May 2012.^5^ Our searches were limited to human studies without language limitation. Studies were included if they (a) evaluated the association of AAM with ovarian cancer risk; (b) presented odds ratio (OR), relative risk (RR), or hazard ratio (HR) estimates with 95% confidence intervals (CI); or had the distribution of cases and total participants (or person‐years for prospective studies) and the OR, RR, or HR with the variance estimates for at least three quantitative exposure categories for studies only providing category results; (c) had at least 60 cases with pathology assessment; (d) had adjusted for covariates related to ovarian cancer. Only studies using the largest number of cases were included if different studies shared the same participants. We also manually searched the references of studies included in our analysis. Details of literature screening are presented in Figure 1A.

If a linear trend was estimated in each included study, it was used directly. For studies only reporting category results, we used the generalized least squares method, assuming linearity of the natural log‐scale estimates of association.^28^ If studies did not use the category with the youngest AAM as the reference, the effective count method was used to recalculate the estimates of association.^29^ The midpoint of each category was assigned to the corresponding risk ratio. When the highest or lowest category was open‐ended, we assumed the width of the category to be the same as that of the adjacent category. Then, we set the reference level to zero and centered each study to the reference level.

We used the nine‐star system of the Newcastle‐Ottawa Scale to conduct the quality assessments of studies (http://www.ohri.ca/programs/clinical_epidemiology/oxford.asp). This system has been developed based on three perspectives (a more detailed description: http://www.ohri.ca/programs/clinical_epidemiology/nosgen.doc): the selection of study groups (a maximum of four stars); the comparability of groups (a maximum of two stars); and the ascertainment of the exposure or outcome for case–control or cohort studies, respectively (a maximum of three stars). A study with quality scores greater than or equal to seven was defined as a high‐quality study.

We included our case–control study in the meta‐analyses. Our case–control study included 289 EOC cases (143 SOC cases) and 206 controls, who had the available information (eg, age, body mass index [BMI], and family history of cancer) from our previous GWAS.^30^ The recruitment information of the participants was described previously.^30^ Briefly, ovarian cancers were newly diagnosed and histologically confirmed from Tianjin Medical University Cancer Hospital. Controls were recruited from cancer‐free subjects and frequency‐matched to cases on age. Demographic characteristics, lifestyles, and family history of cancer were acquired from face‐to‐face interviews by trained interviewers. We used unconditional logistic regression to estimate the association of AAM with EOC risk, adjusting for age, BMI, and family history of cancer. BMI was calculated by dividing a person’s weight (in kilograms) by the square of her height (in meters). Family history of cancer was defined as one or more in first‐ or second‐degree relatives with any cancer.

We performed meta‐analyses based on study designs. The subgroup analyses were based on participants’ ancestry. Participants’ ancestry consisted of Asian, European, and other ancestries. Other ancestry meant participants in the study were non‐Asian and non‐European ancestry, or that participants’ ancestry was not indicated in the study. The Cochran's *Q* and *I* ^2 ^statistics were used to test for heterogeneity and to quantify heterogeneity across studies, respectively. We used the random‐effect model to calculate the summary estimate if heterogeneity existed across studies. Otherwise, the fix‐effect model was used. Egger’s linear regression, Begg’s rank correlation methods, and funnel plots were used to assess potential publication bias. Sensitivity analyses were carried out to evaluate the robustness of the results by excluding one study at a time. Analyses were performed using R v3.3.1 and STATA v13.0. All tests were two‐sided with *P* < 0.05 considered statistically significant, except for heterogeneity test (*P* < 0.1) and publication bias (*P* < 0.1) in meta‐analyses.

### 2.2 Two‐sample MR analyses

#### 2.2.1 MR analyses for the Chinese population

Due to genetic disparity, instrumental variables for Chinese and Europeans were constructed separately. Figure 1B,C show the overview of the MR study. We searched for AAM GWAS performed in Chinese population and found four articles.^16^, ^17^, ^18^, ^19^ One of them was excluded because of incomplete data.^18^ In the remaining three articles, we found 30 nominally replicated GWAS‐identified SNPs (*P* < 0.05). Among them, three SNPs (rs13357391, rs10054991, rs314280) were excluded because of high linkage disequilibrium (*r* ^2^ > 0.6) with another SNP included. Then, we excluded two SNPs associated with ovarian cancer‐related risk factors in Asians (rs10938397 associated with BMI, rs7759938 associated with body height) by searching the GWAS Catalog.^31^ Finally, a total of 25 SNPs were selected as instrumental variables in MR analyses for the Chinese population. We extracted the estimation of their associations with AAM from the published articles (Table S1).^16^, ^17^, ^19^

We calculated the association between AAM‐associated SNPs and EOC risk using our previous GWAS data including 1044 EOC (594 SOC) patients and 1172 age‐matched healthy controls.^30^ Genotyping of genetic variants was performed using Illumina HumanOmniZhongHua‐8 BeadChip. The criteria of quality control for genotyped data were described previously.^30^ Briefly, SNPs were excluded if they had a low call rate (<95%), a low minor allele frequency (MAF) (<0.05), or were deviated from Hardy‐Weinberg equilibrium (*P* < 1 × 10^−5^); individuals were excluded if they had a low call rate (<95%), sex discrepancies between records and data genetically inferred, or if they were relatives or population outliers. We imputed the whole genome data into the 1000 Genomes phase 1 reference panel by IMPUTE2 and SHAPEIT.^32^, ^33^ We performed the association analyses between AAM‐associated SNPs and EOC (SOC) risk using SNPTEST v2.5.2.^34^ Among 25 SNPs used as instrumental variables, seven were genotyped (MAF > 0.05) and the rest were imputed with high quality (Info‐score > 0.9, MAF > 0.05).

#### 2.2.2 MR analyses for the European population

We searched for AAM GWAS performed in Europeans, and selected 432 AAM‐associated SNPs (*P* < 5 × 10^−8^) in two studies.^14^, ^15^ After excluding 31 SNPs because of high linkage disequilibrium (*r* ^2^ > 0.6) with another SNP included and nine SNPs associated with ovarian cancer‐related risk factors identified in the GWAS Catalog,^31^ 392 SNPs were selected as instrumental variables for the European population. The estimation of associations between 392 SNPs and EOC (SOC) risk in the European population was extracted from summary statistics of meta‐analyses by the Ovarian Cancer Association Consortium and Consortium of Investigators of Modifiers of BRCA1/2 studies including 29 396 cases and 68 502 controls (http://ocac.ccge.medschl.cam.ac.uk).^35^ Among the 392 SNPs, 2 SNPs (rs12845465 and rs78472503) had no information in the summary statistics. As a result, a total of 390 SNPs were used as instrumental variables in MR analyses for the European population (Table S2).

The two‐sample MR analyses were conducted using the package "MendelianRandomization" v0.2.2.^36^ Whether the estimates of the associations needed in MR analyses were between SNPs and AAM or between SNPs and ovarian cancer risk, the effect alleles meant later AAM. For SNPs C/G or A/T, the frequency of the effect alleles of the estimates of the associations from different studies was both above 0.5 or both below 0.5, which ensured consistency of the effect alleles. We estimated the association between instrumental variables and outcome by inverse‐variance weighted (IVW)‐based methods. We used both generalized linear regression and robust regression to test the robustness of the association. We also did MR analyses by penalizing the contribution to the analysis of genetic variants with heterogeneous estimates. We performed sensitivity analyses by MR‐Egger and median‐based methods.

### 2.3 Additional analyses for the MR study

In our study, the pleiotropic effects meant that AAM‐associated SNPs were also associated with other ovarian cancer‐related risk factors (eg, BMI, height, birth weight, waist circumference, age, and family history of cancer), or directly associated with ovarian cancer risk. We constructed a weighted genetic score (wGS) using the 25 SNPs to assess the pleiotropic effects in MR analyses in Chinese. The wGS was constructed with the formula 37:

$$\text{wGS}=\beta \left(\sum_{i=1}^{25}\beta_{i}{\text{SNP}}_{i}\right),$$

where β is the mean of the coefficient of 25 SNPs for AAM, β*_i_* is the coefficient of the ith SNP for AAM, and SNP*_i_* is the dosage of the effect alleles of the ith SNP. Individual‐level genetic data were extracted from our previous EOC GWAS.^30^ All effect alleles meant later AAM. The association of wGS with age, BMI, and family history of cancer was performed in the univariate linear regression model to test potential confounder bias of instrumental variables. The pleiotropy was also evaluated by the MR‐Egger intercept.

The strength of instrumental variables in MR analyses for the Chinese population was evaluated by *F* statistic. *F* statistic was calculated with the formula 38:

$$F=\frac{R^{2}\left(n-1-k\right)}{\left(1-R^{2}\right)k},$$

where *R* ^2^ is the phenotypic variation of AAM explained by variants, *n* is the sample size, and *k* is the number of variants. We calculated *R* ^2^ using the individual‐level genetic data from previous GWAS.^30^ In general, an *F* statistic over 10 indicates strong instrumental variables.

Analyses were performed using R v3.3.1. All tests were two sided with *P* < 0.05 considered statistically significant.

## 3 RESULTS

### 3.1 Meta‐analyses of association between AAM and ovarian cancer risk

A total of 26 studies including 11 410 cases and 1 163 117 noncases were included in meta‐analyses.^6^, ^7^, ^8^, ^39^, ^40^, ^41^, ^42^, ^43^, ^44^, ^45^, ^46^, ^47^, ^48^, ^49^, ^50^, ^51^, ^52^, ^53^, ^54^, ^55^, ^56^, ^57^, ^58^, ^59^, ^60^ The quality scores of the included studies ranged from six to nine with a median score of seven. There were 19 high‐quality studies. Table S3 details the characteristics of these studies.

**Figure 2:**
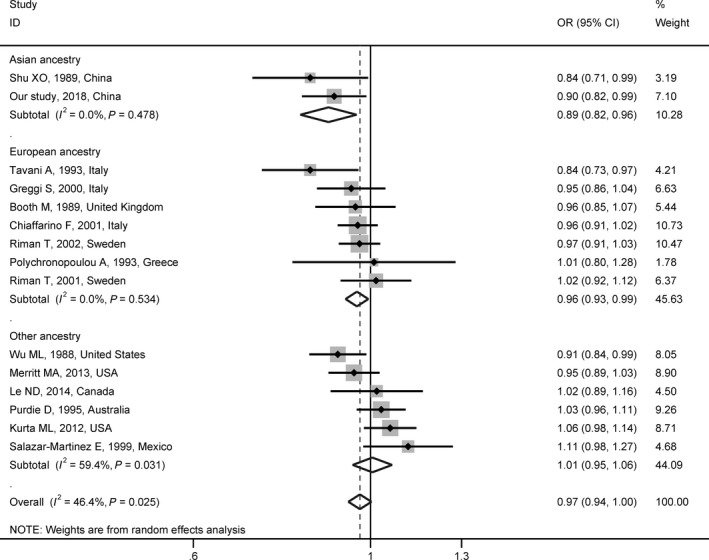
Meta‐analyses of associations between age at menarche and epithelial ovarian cancer risk in case–control studies. This figure contains the estimate of the association between per year older age at menarche and epithelial ovarian cancer risk in each case–control study. The random‐effect model was used to calculate the summary estimate. The subgroup analysis was based on participants’ ancestry. *I* ^2^ and *P* were used to quantify and test for heterogeneity across studies. Abbreviations: OR, odds ratio; CI, confidence interval

The results of meta‐analyses for EOC are as follows:

Among 26 studies, 20 studies were limited to EOC.^6^, ^7^, ^8^, ^39^, ^41^, ^42^, ^43^, ^44^, ^45^, ^46^, ^47^, ^48^, ^51^, ^53^, ^54^, ^55^, ^56^, ^57^, ^58^ In the meta‐analysis of case–control studies with random‐effect model, the pooled OR of the association between per year older AAM and EOC risk was 0.97 (95% CI = 0.94 to 1.00, *P* = 0.071) with evidence of heterogeneity across studies (*P*__het_ = 0.025, *I* ^2^ = 46.4%) (Figure 2). The ORs ranged from 0.96 (95% CI = 0.93 to 0.99, *P* = 0.019) to 0.98 (95% CI = 0.95 to 1.01, *P* = 0.133) in sensitivity analyses (Figure S1A). No publication bias was observed with Egger’s test (*P*‐value for bias = 0.624) or with Begg’s test (*P*‐value for bias = 0.553) or in the funnel plot (Figure S1B). In the subgroup analysis by participants’ ancestry, the pooled OR was 0.89 (95% CI = 0.82 to 0.96, *P* = 0.003) for Asians and 0.96 (95% CI = 0.93 to 0.99, *P* = 0.019) for Europeans, respectively (Figure 2).

**Figure 3:**
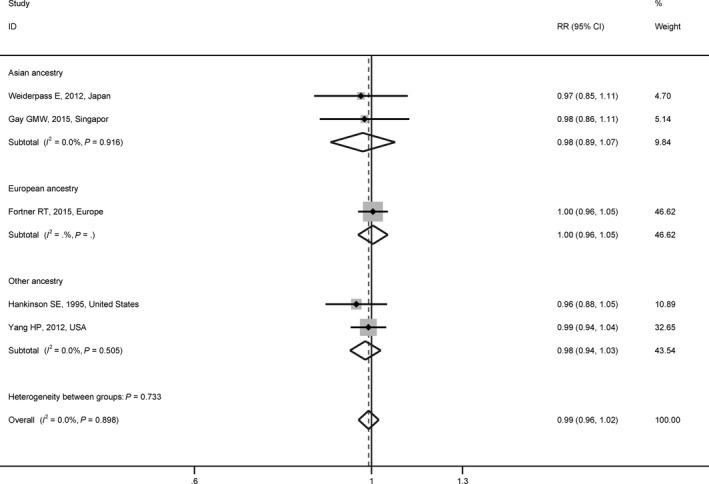
Meta‐analyses of associations between age at menarche and epithelial ovarian cancer risk in cohort studies. This figure contains the estimate of the association between per year older age at menarche and epithelial ovarian cancer risk in each cohort study. The fix‐effect model was used to calculate the summary estimate. The subgroup analysis was based on participants’ ancestry. *I* ^2^ and *P* were used to quantify and test for heterogeneity across studies. Abbreviations: RR, relative risk; CI, confidence interval

In the meta‐analysis of cohort studies with fix‐effect model, the pooled RR of the association between per year older AAM and EOC risk was 0.99 (95% CI = 0.96 to 1.02, *P* = 0.574) without evidence of heterogeneity across studies (*P*__het_ = 0.898, *I* ^2^ = 0.0%) (Figure 3). The RRs ranged from 0.98 (95% CI = 0.94 to 1.02, *P* = 0.347) to 1.00 (95% CI = 0.97 to 1.03, *P* = 0.798) in sensitivity analyses (Figure S1C). No publication bias was observed with Egger’s test (*P*‐value for bias = 0.129) or with Begg’s test (*P*‐value for bias = 0.221) or in the funnel plot (Figure S1D). In the subgroup analysis by participants’ ancestry, the pooled RR was 0.98 (95% CI = 0.89 to 1.07, *P* = 0.596) for Asians and 1.00 (95% CI = 0.96 to 1.05, *P* = 0.855) for Europeans, respectively (Figure 3).

The results of meta‐analyses for all subtypes’ ovarian cancer and SOC are as follows:

In the meta‐analysis of case–control studies with random‐effect model, the pooled OR of the association between per year older AAM and ovarian cancer risk was 0.96 (95% CI = 0.93 to 1.00, *P* = 0.036) with evidence of heterogeneity across studies (*P*__het_ = 0.003, *I* ^2^ = 54.0%) (Figure S1E). For cohort studies, the pooled RR in fix‐effect model was 0.99 (95% CI = 0.96 to 1.01, *P* = 0.366) without evidence of heterogeneity across studies (*P*__het_ = 0.916, *I* ^2^ = 0.0%) (Figure S1E). For SOC, a total of 5 studies were included,^54^, ^57^, ^58^, ^60^ and no association was observed either in case–control or cohort studies with fix‐effect model (Figure S1F).

### 3.2 Two‐sample MR analyses of association between AAM and EOC risk

#### 3.2.1 The Chinese population

Among 25 SNPs used as instrumental variables, none of them were associated with ovarian cancer‐related risk factors in Asians by searching the GWAS Catalog. Additionally, no association was observed between wGS and age, BMI, or family history of cancer (Table S4). The instrumental variables could explain about 13.9% of the variance in AAM. The* F *statistic for combined SNPs was 14.1, which suggested our instrument was unlikely to suffer from weak instrument bias.

In two‐sample MR analyses, the OR of EOC risk per year increase in genetically predicted AAM was 0.81 (95% CI = 0.67 to 0.97, *P* = 0.026) by IVW methods. In sensitivity analyses, the results by MR‐Egger and median‐based methods were similar to those by IVW‐based methods. Although the median‐based method indicated no association, the direction of the association was consistent and the OR was similar. The MR‐Egger intercept suggested that pleiotropy might not influence the results (MR‐Egger intercept *P* > 0.05). AAM was also negatively associated with SOC risk. Table 1 shows the results.

**Table 1:** Association of genetically predicted per year older age at menarche and epithelial (serous) ovarian cancer risk using GWAS summarized data in Chinese population

MR methods	EOC		SOC	
	OR (95% CI)	*P*	OR (95% CI)	*P*
IVW	0.81 (0.67 to 0.97)	0.026	0.76 (0.62 to 0.94)	0.010
Penalized IVW	0.80 (0.67 to 0.95)	0.010	0.75 (0.63 to 0.89)	0.001
Robust IVW	0.79 (0.69 to 0.89)	<0.001	0.75 (0.65 to 0.85)	<0.001
Penalized robust IVW	0.78 (0.70 to 0.88)	<0.001	0.74 (0.65 to 0.84)	<0.001
MR‐Egger	0.73 (0.58 to 0.90)	0.004	0.69 (0.54 to 0.88)	0.003
(intercept)	0.04 (−0.01 to 0.07)^a^	0.088	0.03 (−0.01 to 0.08)^a^	0.152
Simple median	0.76 (0.47 to 1.23)	0.259	0.69 (0.40 to 1.19)	0.184

	*P*__het_	*I* ^2^	*P*__het_	*I* ^2^
Tests for heterogeneity	0.029	38.1%	0.082	29.8%

Abbreviations: CI, confidence interval; EOC, epithelial ovarian cancer; GWAS, genome‐wide association studies; IVW, inverse‐variance weighted; MR, Mendelian randomization; OR, odds ratio; SOC, serous ovarian cancer.

a Means beta instead of OR.

#### 3.2.2 The European population

Among 390 SNPs used as instrumental variables, none of them were associated with ovarian cancer‐related risk factors in Europeans by searching the GWAS Catalog. In two‐sample MR analyses, the OR of EOC risk per year increase in genetically predicted AAM was 0.94 (95% CI = 0.90 to 0.98, *P* = 0.003) by IVW methods. In sensitivity analyses, the results by MR‐Egger and median‐based methods were similar to those by IVW‐based methods. The MR‐Egger intercept suggested that pleiotropy might not influence the results (MR‐Egger intercept *P* >  0.05). AAM was also negatively associated with SOC risk. Table 2 shows the results.

**Table 2:** Association of genetically predicted per year older age at menarche and epithelial (serous) ovarian cancer risk using GWAS summarized data in European population

MR methods	EOC		SOC	
	OR (95% CI)	*P*	OR (95% CI)	*P*
IVW	0.94 (0.90 to 0.98)	0.003	0.95 (0.91 to 1.00)	0.035
Penalized IVW	0.94 (0.90 to 0.98)	0.004	0.96 (0.91 to 1.00)	0.044
Robust IVW	0.94 (0.90 to 0.98)	0.007	0.95 (0.91 to 1.00)	0.051
Penalized robust IVW	0.94 (0.90 to 0.98)	0.007	0.96 (0.91 to 1.00)	0.051
MR‐Egger	0.96 (0.85 to 1.09)	0.524	0.99 (0.86 to 1.13)	0.864
(intercept)	−0.00 (−0.01 to 0.00)^a^	0.678	−0.00 (−0.01 to 0.00)^a^	0.555
Simple median	0.97 (0.91 to 1.03)	0.327	0.98 (0.92 to 1.05)	0.533

	*P*__het_	*I* ^2^	*P*__het_	*I* ^2^
Tests for heterogeneity	3E‐04	21.1%	0.001	19.2%

Abbreviations: CI, confidence interval; EOC, epithelial ovarian cancer; GWAS, genome‐wide association studies; IVW, inverse‐variance weighted; MR, Mendelian randomization; OR, odds ratio; SOC, serous ovarian cancer.

a Means beta instead of OR.

## 4 DISCUSSION

In this study, after systematic meta‐analyses, we performed two‐sample MR analyses to evaluate the causal effect of AAM in EOC (SOC) etiology. In meta‐analyses of case–control studies, we observed an inverse association between per year older AAM and ovarian cancer risk. However, no association was observed in cohort studies. In our MR study using the IVW method, we observed a 19% lower risk of EOC per year increase in AAM (OR = 0.81, 95% CI = 0.67 to 0.97, *P* = 0.026) and a 24% lower risk of SOC per year increase in AAM (OR = 0.76, 95% CI = 0.62 to 0.94, *P* = 0.010) in Chinese, and a 6% lower risk of EOC per year increase in AAM (OR = 0.94, 95% CI = 0.90 to 0.98, *P* = 0.003) and a 5% lower risk of SOC per year increase in AAM (OR = 0.95, 95% CI = 0.91 to 1.00, *P* = 0.035) in Europeans.

In meta‐analyses, the results of case–control studies were different from that of cohort studies, which might be caused by different covariates in each study and different heterogeneity which was only detected in case–control studies. Meanwhile, it should be noted that the effect size was similar between case–control (OR = 0.96) and cohort (RR = 0.99) studies. The estimates of associations were weaker in meta‐analyses than in MR analyses. The estimate of MR studies reflects the effects of lifelong interference, whereas that of observational studies reflects more acute effects.^61^ Also, observational studies may suffer from measurement error and confounding bias. In meta‐analyses, we excluded many studies because of unusable data. Hence, the limited sample size might reduce the statistical power. Results show the weaker association in Europeans than in Asians. The previous meta‐analysis by category^5^ attributed this difference to the later mean AAM in Asians than that in Europeans. We agreed with this opinion. Furthermore, AAM was determined by puberty hormonal levels.^4^ Different hormone levels among various races and ethnics may lead to the difference.^62^, ^63^

AAM appears to have a significant genetic component.^4^ Familial and twin studies suggested a substantial proportion of genetic influences on AAM.^13^, ^64^ Previous MR studies have suggested that AAM may be causally associated with risk of breast, endometrial, and prostate cancers.^15^, ^65^ Although its sensitivity tests suggested a possible pleiotropic effect, the previous MR analysis indicated an inverse association between AAM and risk of ovarian cancer.^15^ And our study provided evidence of a connection between AAM and EOC risk. Breast, prostate, endometrial, and ovarian cancers are all hormone‐related diseases. AAM can reflect pubertal hormonal levels. The HPG axis plays a critical role in the secretion and regulation of hormones. Therefore, genes and pathways on the HPG axis are related to both AAM and ovarian cancer. It has been reported that *KiSS1*, *GNRHR*, and *DUSP6* on the HPG axis are involved in the development of ovarian cancer.^66^, ^67^, ^68^, ^69^ Onset of puberty needs the activation of the GnRH secretory system. GnRH signaling, which plays a key role in the HPG axis,^70^ may influence ovarian cancer risk via various mechanisms.^71^, ^72^, ^73^ Rs2153127, as one of instrumental variables of AAM in our MR study, is indicated to be associated with the expression of *LIN28B* in pituitary (https://www.gtexportal.org/home/snp/rs2153127). *LIN28B* was reported to be related to the proliferation, migration, and apoptosis of ovarian cancer.^74^, ^75^ Further studies on variants associated with AAM or in the HPG axis may be beneficial to identify additional genetic variants for ovarian cancer risk. Additionally, the negative association between AAM and EOC risk could be explained by hormone‐related “incessant ovulation” hypothesis, which supported increasing numbers of lifetime ovulations with higher risk of ovarian cancer.^2^

Our study has several strengths and limitations. One of the strengths is that our study was performed in two independent populations, which makes the conclusion be generalized to a broader population. Additionally, three assumptions of MR studies were basically satisfied (assumption one: the instrumental variables are associated with the exposure; assumption two: the instrumental variables are independent of the confounding factors that confound the association of the exposure and the outcome; assumption three: the instrumental variables are independent of the outcome given the exposure and the confounding factors). For the MR analyses in Chinese, the instrumental variables were selected from AAM GWAS in Chinese at *P* < 0.05, and the *F* statistic was above 10 (assumption one); No association was observed between wGS constructed by instrumental variables and age, BMI, or family history of cancer, and none of instrumental variables were associated with other ovarian cancer‐related risk factors in Asians by searching the GWAS Catalog, and the MR‐Egger intercept indicated that pleiotropic effects might not be a problem (assumption two); none of instrumental variables were associated with ovarian cancer risk in Asians by searching the GWAS Catalog (assumption three). For the MR analyses in Europeans, the instrumental variables were selected from AAM GWAS in Europeans at *P* < 5 × 10^−8^ (assumption one); none of instrumental variables were associated with other ovarian cancer‐related risk factors in Europeans by searching the GWAS Catalog, and the MR‐Egger intercept indicated that pleiotropic effects might not be a problem (assumption two); none of instrumental variables were associated with ovarian cancer risk in Europeans by searching the GWAS Catalog (assumption three). There are several limitations in our study. Firstly, the threshold of *P*‐value of SNPs we selected as instrumental variables in Chinese was 0.05 rather than 5 × 10^−8^, because only rs7759938 (also associated with body height) reached the genome‐wide significant threshold. Notably, most of these selected SNPs have been validated in the European population (*P* < 5 × 10^−8^).^14^, ^16^, ^20^, ^21^, ^22^, ^23^, ^24^ It is expected that more AAM‐associated SNPs could be found and that stronger instrumental variables could be utilized in further studies. Secondly, the sample size of our MR study in Chinese population was small, and a larger one is needed. Thirdly, we did not perform analyses in mucinous, endometrioid, or clear cell ovarian cancer due to small sample sizes in each subtype.

In summary, our study provides evidence that AAM may be a causal risk factor of EOC. The underlying mechanisms remain to be elucidated.

## CONFLICT OF INTEREST

None declared.

## Data Availability

The data that support the findings of this study are available from the corresponding author upon reasonable request.
