# Supplementary material for: Age at menarche and epithelial ovarian cancer risk: A meta‐analysis and Mendelian randomization study
Source: Cancer Med. 2019 May 30;8(8):4012–22. doi: 10.1002/cam4.2315 (PMC6639189; doi:10.1002/cam4.2315)
Supplement: Supplementary file 1 [file CAM4-8-4012-s001.pdf]

Supplementary Table 1. Characteristics of SNPs used in Mendelian randomization analyses of Chinese population

PMID	SNP	Chr	Position†	Effect_allele	Reference_allele	OR (95% CI)	*P*
27629107	rs2274465	1	44121557	C	G	1.06 (1.01 to 1.11)	1.40E-02
27629107	rs466639	1	165394882	C	T	1.06 (1.00 to 1.12)	4.00E-02
27629107	rs1400974	2	199638690	A	G	1.08 (1.04 to 1.13)	3.30E-04
27629107	rs11715566	3	117562436	T	C	1.08 (1.03 to 1.12)	7.00E-04
27629107	rs1023935	4	35150284	C	T	1.16 (1.09 to 1.23)	4.90E-06
27629107	rs3733631	4	104641103	C	G	1.05 (1.01 to 1.10)	2.40E-02
19282985	rs1859345	5	136447420	T	C	2.21 (1.32 to 3.69)	4.37E-03
19282985	rs17779700	5	136572793	A	G	2.00 (1.04 to 3.84)	2.20E-02
27629107	rs17171818	5	137725003	C	T	1.04 (1.00 to 1.09)	4.50E-02
27629107	rs2153127	6	105348544	T	C	1.07 (1.02 to 1.11)	4.00E-03
23508249	rs314277	6	105407662	A	C	1.20 (1.02 to 1.40)	3.04E-02
27629107	rs10816359	9	108757670	T	G	1.06 (1.01 to 1.11)	2.60E-02
27629107	rs10453225	9	108920220	G	T	1.08 (1.04 to 1.13)	2.40E-04
27629107	rs10739221	9	109060830	C	T	1.05 (1.00 to 1.09)	4.30E-02
27629107	rs10980921	9	114279912	C	T	1.06 (1.01 to 1.12)	2.60E-02
27629107	rs1874984	10	1731871	C	G	1.05 (1.00 to 1.09)	3.50E-02
27629107	rs79195475	10	66964843	C	T	1.13 (1.07 to 1.18)	3.40E-06
27629107	rs2063730	11	78048524	C	A	1.05 (1.01 to 1.10)	2.10E-02
27629107	rs10895140	11	101436721	G	A	1.06 (1.02 to 1.11)	5.00E-03
27629107	rs11215400	11	115052635	C	A	1.07 (1.00 to 1.13)	4.20E-02
27629107	rs12915845	15	89042467	C	T	1.07 (1.00 to 1.13)	3.80E-02
27629107	rs246185	16	14395432	C	T	1.06 (1.02 to 1.11)	7.00E-03
27629107	rs1129700	16	29918034	T	C	1.08 (1.03 to 1.14)	2.00E-03
27629107	rs12607903	18	3817134	C	T	1.07 (1.02 to 1.12)	3.00E-03
27629107	rs652260	19	7900562	T	C	1.05 (1.01 to 1.10)	2.00E-02

† means that the chromosome physical position is based on the National Center for Biotechnology Information database, Build 37; PMID = PubMed Unique Identifier; SNP = single nucleotide polymorphism; Chr = chromosome; OR = odds ratio; CI = confidence interval.

Supplementary Table 2. Characteristics of SNPs used in Mendelian randomization analyses of Europeans

PMID	SNP	Chr	Position†	Effect_allele	Reference_allele	OR (95% CI)	*P*
28436984	rs2308069	1	7419312	C	CTG	1.03 (1.02 to 1.04)	6.02E-10
28436984	rs6678140	1	8436802	C	T	1.03 (1.02 to 1.04)	7.54E-11
28436984	rs34646499	1	14138827	C	CG	1.04 (1.02 to 1.05)	5.08E-10
28436984	rs12125335	1	21385436	C	T	1.05 (1.04 to 1.06)	2.33E-15
28436984	rs141847393	1	27212209	T	C	1.04 (1.03 to 1.05)	2.96E-08
28436984	rs360495	1	33228197	A	T	1.04 (1.03 to 1.06)	2.89E-09
28436984	rs4970598	1	38940215	T	C	1.06 (1.04 to 1.09)	2.34E-08
28436984	rs11209331	1	41456689	T	C	1.02 (1.02 to 1.03)	9.83E-10
28436984	rs11210871	1	44029353	C	G	1.04 (1.03 to 1.05)	3.07E-21
25231870	rs2274465	1	44121557	C	G	1.03 (1.02 to 1.04)	2.00E-09
28436984	rs643428	1	54728858	C	T	1.02 (1.01 to 1.03)	3.21E-08
28436984	rs11357467	1	65822268	T	TA	1.03 (1.02 to 1.03)	1.04E-09
28436984	rs7516763	1	65972550	A	C	1.02 (1.02 to 1.03)	1.49E-09
28436984	rs11209943	1	72750500	A	G	1.04 (1.03 to 1.05)	1.08E-22
28436984	rs1040070	1	74977870	C	G	1.05 (1.04 to 1.06)	7.01E-38
28436984	rs10782777	1	82562929	G	A	1.03 (1.02 to 1.03)	8.40E-10
28436984	rs7517629	1	91196099	G	A	1.02 (1.02 to 1.03)	3.10E-09
28436984	rs11165924	1	98375448	A	G	1.03 (1.02 to 1.04)	4.83E-14
28436984	rs4561063	1	102520898	T	G	1.03 (1.02 to 1.04)	7.89E-16
28436984	rs61817552	1	150423577	G	A	1.03 (1.02 to 1.04)	2.42E-09
28436984	rs6661100	1	150758727	T	C	1.05 (1.03 to 1.06)	2.18E-12
28436984	rs4845364	1	154141908	A	G	1.02 (1.01 to 1.03)	1.62E-08
28436984	rs9427116	1	154631123	T	C	1.02 (1.01 to 1.03)	4.24E-08
28436984	rs2343507	1	162895515	A	C	1.02 (1.02 to 1.03)	2.88E-09
28436984	rs2661339	1	163018934	T	G	1.05 (1.04 to 1.07)	1.34E-09
28436984	rs157877	1	165398744	G	A	1.09 (1.08 to 1.10)	2.25E-48
28436984	rs7542538	1	165426193	T	C	1.03 (1.02 to 1.04)	7.63E-09
28436984	rs61826838	1	174031021	C	T	1.03 (1.02 to 1.04)	2.34E-09
28436984	rs506589	1	177894287	T	C	1.07 (1.06 to 1.08)	1.54E-47
28436984	rs61828391	1	179732142	G	A	1.03 (1.02 to 1.04)	4.31E-08
28436984	rs58989029	1	199798561	G	GA	1.03 (1.02 to 1.04)	7.63E-16
28436984	rs11240695	1	204158132	C	A	1.03 (1.02 to 1.04)	3.06E-14
28436984	rs4951261	1	205717823	A	C	1.03 (1.02 to 1.04)	5.36E-12
28436984	rs12040029	1	213451958	C	T	1.04 (1.03 to 1.05)	4.04E-10
28436984	rs62104180	2	466003	A	G	1.12 (1.10 to 1.14)	3.98E-29
28436984	rs7576624	2	625029	C	T	1.08 (1.07 to 1.09)	4.98E-50
28436984	rs7587651	2	10368606	C	T	1.02 (1.02 to 1.03)	4.26E-09
28436984	rs150821390	2	24106445	T	C	1.07 (1.05 to 1.10)	6.59E-09
28436984	rs11461653	2	25159399	G	GC	1.03 (1.02 to 1.03)	6.58E-10
28436984	rs72787511	2	32816089	C	G	1.07 (1.04 to 1.09)	1.38E-08
28436984	rs10175423	2	42970161	C	T	1.03 (1.02 to 1.03)	4.25E-09

28436984	rs17390720	2	44952254	C	G	1.03 (1.02 to 1.04)	3.70E-09
28436984	rs149544395	2	48013858	C	CT	1.03 (1.02 to 1.04)	1.65E-11
25231870	rs6747380	2	56587749	A	G	1.07 (1.06 to 1.09)	6.00E-28
28436984	rs1025128	2	60175475	G	C	1.02 (1.01 to 1.03)	1.88E-08
28436984	rs10205969	2	61367664	C	T	1.04 (1.03 to 1.05)	5.62E-13
28436984	rs12467441	2	61685826	C	T	1.04 (1.03 to 1.05)	8.99E-12
28436984	rs2723065	2	65279414	G	A	1.03 (1.02 to 1.03)	2.59E-10
28436984	rs2312205	2	69704941	A	G	1.03 (1.02 to 1.04)	1.26E-09
28436984	rs34437050	2	73535526	A	G	1.27 (1.22 to 1.32)	4.04E-32
28436984	rs2679894	2	105870779	A	G	1.05 (1.04 to 1.06)	5.37E-33
28436984	rs2558101	2	137613322	G	A	1.02 (1.02 to 1.03)	4.28E-08
28436984	rs35935052	2	142302503	T	G	1.04 (1.03 to 1.06)	5.01E-16
28436984	rs6434162	2	153556801	G	A	1.04 (1.03 to 1.05)	2.51E-12
28436984	rs142058842	2	156621725	G	C	1.07 (1.06 to 1.08)	2.42E-40
25231870	rs17236969	2	156752459	T	C	1.05 (1.03 to 1.07)	3.00E-09
28436984	rs145438026	2	157228255	C	T	1.07 (1.06 to 1.09)	2.34E-18
28436984	rs72186109	2	164538904	C	CACA	1.03 (1.02 to 1.04)	4.93E-13
28436984	rs2271758	2	172701157	G	T	1.02 (1.01 to 1.03)	3.96E-08
28436984	rs842567	2	184291116	C	A	1.03 (1.02 to 1.04)	9.38E-12
28436984	rs10931831	2	199621641	C	T	1.05 (1.05 to 1.06)	1.87E-39
25231870	rs17233066	2	199644038	C	T	1.09 (1.07 to 1.12)	6.00E-11
28436984	rs13023912	2	199756278	G	A	1.05 (1.04 to 1.06)	4.82E-36
28436984	rs16841867	2	203168235	C	G	1.05 (1.03 to 1.06)	2.56E-14
28436984	rs184033703	2	206956138	G	A	1.05 (1.03 to 1.07)	1.85E-08
28436984	rs138546321	2	209607647	T	TTA	1.06 (1.05 to 1.07)	3.01E-18
28436984	rs6735626	2	213403972	A	G	1.02 (1.01 to 1.03)	2.96E-08
28436984	rs73820560	3	1906245	C	A	1.03 (1.02 to 1.04)	1.68E-08
28436984	rs9867904	3	18442437	G	C	1.03 (1.02 to 1.04)	3.42E-12
28436984	rs73035994	3	24206463	C	T	1.09 (1.07 to 1.12)	5.22E-15
28436984	rs1984870	3	24715135	T	G	1.04 (1.04 to 1.05)	5.61E-27
28436984	rs77955256	3	44883523	T	A	1.04 (1.02 to 1.05)	7.10E-09
25231870	rs7647973	3	49510931	A	G	1.05 (1.04 to 1.06)	1.00E-16
28436984	rs115435316	3	49568181	A	G	1.12 (1.10 to 1.15)	1.67E-24
28436984	rs6445624	3	51358019	A	G	1.04 (1.03 to 1.05)	6.41E-14
28436984	rs10933	3	52719816	C	T	1.02 (1.02 to 1.03)	6.72E-10
28436984	rs7431217	3	68595634	T	C	1.02 (1.02 to 1.03)	6.19E-09
28436984	rs7426534	3	84462073	G	A	1.02 (1.02 to 1.03)	2.39E-08
28436984	rs9758500	3	86910329	G	A	1.05 (1.04 to 1.05)	1.36E-30
28436984	rs709488	3	107700952	C	A	1.02 (1.01 to 1.03)	1.38E-08
28436984	rs9834893	3	114574749	G	C	1.05 (1.04 to 1.07)	9.41E-12
28436984	rs10934420	3	117552111	C	T	1.06 (1.05 to 1.06)	1.54E-47
25231870	rs2687729	3	127895226	G	A	1.04 (1.03 to 1.05)	1.00E-10
25231870	rs2600959	3	132615464	A	G	1.04 (1.03 to 1.05)	4.00E-11
28436984	rs6439713	3	137128815	A	C	1.03 (1.02 to 1.03)	2.13E-10

28436984	rs11711674	3	156532953	T	C	1.02 (1.01 to 1.03)	1.77E-08
28436984	rs582780	3	172121443	A	G	1.03 (1.02 to 1.03)	1.41E-11
28436984	rs7649124	3	184030827	C	G	1.03 (1.02 to 1.04)	2.00E-13
28436984	rs2300922	3	185651469	T	C	1.04 (1.04 to 1.05)	1.11E-28
28436984	rs2108753	4	3266860	T	C	1.03 (1.02 to 1.04)	1.20E-13
25231870	rs1038903	4	28752054	T	C	1.04 (1.03 to 1.05)	2.00E-11
28436984	rs144582188	4	45165650	A	ATTC	1.05 (1.04 to 1.06)	1.50E-27
28436984	rs4588499	4	45910674	G	A	1.02 (1.02 to 1.03)	1.47E-09
28436984	rs202110205	4	60215498	C	CAG	1.03 (1.02 to 1.04)	4.99E-08
28436984	rs3113862	4	95143122	G	A	1.04 (1.03 to 1.05)	9.69E-22
28436984	rs55784701	4	104247262	T	C	1.03 (1.02 to 1.04)	1.08E-08
28436984	rs3733632	4	104640935	G	A	1.06 (1.04 to 1.07)	1.04E-24
28436984	rs62342064	4	104665972	T	C	1.06 (1.04 to 1.07)	4.51E-18
28436984	rs115260227	4	104774698	G	A	1.18 (1.12 to 1.23)	1.90E-11
28436984	rs17035311	4	106066293	A	C	1.04 (1.03 to 1.05)	2.26E-11
28436984	rs62316795	4	132621869	A	C	1.04 (1.03 to 1.05)	4.98E-11
28436984	rs13120031	4	177465182	T	C	1.03 (1.02 to 1.04)	1.57E-11
28436984	rs10521021	5	35030311	G	T	1.02 (1.02 to 1.03)	4.77E-09
28436984	rs62361685	5	41994067	T	C	1.06 (1.04 to 1.07)	3.09E-10
28436984	rs7712046	5	43134968	C	T	1.03 (1.03 to 1.04)	9.42E-16
28436984	rs813301	5	52909927	T	C	1.03 (1.02 to 1.04)	2.84E-12
28436984	rs256350	5	59140876	C	T	1.02 (1.02 to 1.03)	2.84E-08
28436984	rs80170948	5	64020316	G	T	1.07 (1.05 to 1.09)	2.80E-10
28436984	rs13173441	5	77048448	T	C	1.03 (1.02 to 1.05)	1.86E-08
28436984	rs17085593	5	95630705	C	G	1.02 (1.02 to 1.03)	3.53E-09
28436984	rs2916578	5	107316227	G	A	1.03 (1.02 to 1.03)	6.99E-10
28436984	rs654354	5	110503301	T	A	1.02 (1.02 to 1.03)	2.36E-09
28436984	rs247520	5	110876057	T	C	1.04 (1.03 to 1.05)	1.99E-15
28436984	rs1566385	5	111130474	A	G	1.06 (1.04 to 1.08)	1.81E-12
28436984	rs62379978	5	133915969	G	T	1.07 (1.05 to 1.08)	6.55E-32
28436984	rs3815212	5	137761555	T	C	1.03 (1.02 to 1.04)	3.21E-13
28436984	rs6878910	5	138281261	A	G	1.04 (1.03 to 1.05)	4.31E-10
28436984	rs975642	5	139384490	C	T	1.02 (1.02 to 1.03)	2.51E-10
28436984	rs71592143	5	141656062	T	A	1.03 (1.02 to 1.04)	1.32E-08
28436984	rs1428120	5	153541904	T	G	1.03 (1.02 to 1.03)	7.72E-11
28436984	rs437836	5	156715068	T	C	1.04 (1.03 to 1.05)	1.15E-11
28436984	rs34371367	5	165937769	A	AT	1.02 (1.02 to 1.03)	2.17E-08
25231870	rs9647570	5	167370263	G	T	1.05 (1.04 to 1.07)	1.00E-11
28436984	rs2546959	5	167404411	T	G	1.03 (1.02 to 1.04)	1.82E-09
28436984	rs4976623	5	167947996	C	G	1.03 (1.02 to 1.04)	1.71E-09
28436984	rs6864818	5	168734867	T	C	1.04 (1.03 to 1.05)	4.67E-15
28436984	rs4701140	5	179034260	A	G	1.02 (1.02 to 1.03)	1.95E-09
28436984	rs2770957	5	180656734	C	G	1.03 (1.02 to 1.04)	5.90E-12
28436984	rs446745	6	14918298	C	T	1.03 (1.02 to 1.04)	2.81E-08

28436984	rs6927679	6	18559687	T	C	1.03 (1.02 to 1.04)	2.52E-10
28436984	rs1539310	6	22562485	A	G	1.02 (1.02 to 1.03)	4.59E-08
28436984	rs12663002	6	28441634	T	C	1.04 (1.03 to 1.06)	6.89E-14
28436984	rs62391851	6	29740548	G	A	1.06 (1.04 to 1.08)	2.44E-12
25231870	rs16896742	6	29922740	G	A	1.04 (1.03 to 1.05)	3.00E-10
28436984	rs3021057	6	32652363	T	C	1.03 (1.02 to 1.03)	2.01E-08
28436984	rs9349203	6	41893323	G	A	1.04 (1.03 to 1.05)	5.93E-25
28436984	rs79541760	6	50930848	A	T	1.04 (1.03 to 1.05)	6.98E-13
28436984	rs222440	6	52946320	C	T	1.03 (1.02 to 1.04)	9.73E-11
28436984	rs9474996	6	54640512	T	A	1.03 (1.03 to 1.04)	3.82E-19
28436984	rs9382676	6	56859084	T	C	1.04 (1.03 to 1.05)	8.77E-16
28436984	rs7753896	6	76347020	A	G	1.03 (1.02 to 1.04)	2.46E-15
28436984	rs7757654	6	77173780	C	T	1.03 (1.02 to 1.04)	2.51E-13
28436984	rs1414186	6	77713859	G	T	1.04 (1.03 to 1.05)	1.99E-19
28436984	rs11756746	6	84286477	A	G	1.03 (1.02 to 1.03)	3.95E-08
25231870	rs9321659	6	100116092	A	G	1.06 (1.04 to 1.08)	3.00E-16
28436984	rs9403051	6	100194846	A	G	1.04 (1.03 to 1.04)	1.76E-21
25231870	rs13196561	6	100760170	C	A	1.04 (1.03 to 1.05)	8.00E-12
28436984	rs12200565	6	100983589	T	C	1.03 (1.02 to 1.04)	1.11E-15
28436984	rs6911407	6	108867031	A	C	1.03 (1.02 to 1.04)	1.22E-13
28436984	rs235696	6	124253495	C	G	1.02 (1.01 to 1.03)	2.66E-08
28436984	rs4897178	6	126727908	T	G	1.04 (1.04 to 1.05)	1.02E-27
25231870	rs6938574	6	128390980	T	C	1.04 (1.03 to 1.06)	2.00E-09
28436984	rs78928932	6	136228617	C	T	1.06 (1.04 to 1.08)	1.06E-09
28436984	rs117530880	6	146687748	G	T	1.07 (1.04 to 1.09)	2.58E-08
28436984	rs6911527	6	148285329	T	C	1.03 (1.02 to 1.04)	3.18E-09
28436984	rs6933660	6	151803754	C	A	1.03 (1.03 to 1.04)	8.99E-17
28436984	rs910425	6	170652191	G	A	1.02 (1.01 to 1.03)	1.63E-08
28436984	rs10268051	7	27763590	A	C	1.03 (1.02 to 1.03)	3.88E-08
28436984	rs17171852	7	41392815	C	A	1.04 (1.03 to 1.05)	3.42E-15
28436984	rs1079866	7	41470093	G	C	1.07 (1.06 to 1.09)	3.65E-37
28436984	rs1470750	7	50576648	G	C	1.02 (1.01 to 1.03)	1.44E-08
28436984	rs2267812	7	74138121	A	C	1.04 (1.03 to 1.05)	1.69E-17
28436984	rs187760798	7	75142551	T	C	1.06 (1.04 to 1.07)	1.10E-15
28436984	rs1030015	7	78139581	G	T	1.02 (1.01 to 1.03)	4.45E-08
28436984	rs149226155	7	93215658	G	A	1.02 (1.02 to 1.03)	5.67E-09
28436984	rs15671	7	94186064	C	A	1.02 (1.02 to 1.03)	1.25E-09
28436984	rs999885	7	99701176	A	G	1.02 (1.02 to 1.03)	2.79E-10
28436984	rs1456031	7	114296102	T	C	1.02 (1.01 to 1.03)	2.44E-08
28436984	rs10237306	7	121955981	T	G	1.03 (1.02 to 1.04)	1.34E-13
25231870	rs11767400	7	122160742	A	C	1.04 (1.03 to 1.05)	4.00E-11
28436984	rs11556924	7	129663496	T	C	1.02 (1.01 to 1.03)	3.66E-08
28436984	rs17563472	7	130409054	C	T	1.06 (1.04 to 1.08)	1.52E-08
28436984	rs12707076	7	132729814	C	G	1.03 (1.02 to 1.04)	4.88E-12

28436984	rs13233916	7	138874416	G	C	1.05 (1.04 to 1.07)	3.11E-11
28436984	rs7004265	8	1523903	T	C	1.02 (1.02 to 1.03)	5.12E-09
28436984	rs2688326	8	3767623	C	T	1.04 (1.03 to 1.05)	4.34E-18
28436984	rs2724961	8	4560227	C	T	1.05 (1.04 to 1.05)	3.76E-33
28436984	rs4875424	8	4831685	C	T	1.03 (1.03 to 1.04)	1.99E-16
28436984	rs6185	8	25280800	G	C	1.03 (1.02 to 1.04)	9.48E-12
28436984	rs13278754	8	34902952	G	C	1.03 (1.02 to 1.03)	2.40E-09
28436984	rs4487799	8	53163528	A	T	1.02 (1.02 to 1.03)	1.09E-08
28436984	rs16918378	8	53877882	T	C	1.05 (1.04 to 1.06)	9.08E-16
28436984	rs56409371	8	53934144	G	A	1.04 (1.03 to 1.05)	1.95E-12
28436984	rs1449543	8	76591987	T	C	1.02 (1.02 to 1.03)	5.06E-09
28436984	rs11786868	8	77653945	C	G	1.03 (1.02 to 1.04)	4.27E-10
28436984	rs10094506	8	78116203	C	T	1.05 (1.04 to 1.06)	2.46E-26
28436984	rs35485457	8	78679087	G	T	1.04 (1.03 to 1.05)	2.65E-17
28436984	rs7465046	8	87319950	C	T	1.04 (1.03 to 1.05)	1.52E-18
28436984	rs2441873	8	105329549	T	G	1.02 (1.02 to 1.03)	1.74E-09
28436984	rs7826872	8	132071766	T	C	1.03 (1.02 to 1.03)	4.26E-11
28436984	rs2542420	8	140645701	C	G	1.03 (1.03 to 1.04)	1.52E-16
25231870	rs1469039	8	140651779	A	G	1.05 (1.04 to 1.07)	4.00E-12
28436984	rs552491	9	1711210	G	A	1.03 (1.02 to 1.04)	1.11E-13
28436984	rs913588	9	7174673	G	A	1.03 (1.03 to 1.04)	6.68E-19
28436984	rs10959016	9	10283451	G	A	1.03 (1.02 to 1.04)	2.21E-10
28436984	rs10959552	9	11130009	G	A	1.04 (1.03 to 1.05)	4.50E-10
28436984	rs1601615	9	11813745	C	T	1.03 (1.02 to 1.04)	7.57E-13
28436984	rs7849973	9	22819576	C	G	1.02 (1.02 to 1.03)	2.82E-09
28436984	rs1329767	9	73798371	C	A	1.03 (1.02 to 1.04)	2.43E-13
28436984	rs146865828	9	75864509	T	TTAAA	1.06 (1.04 to 1.07)	3.24E-12
28436984	rs2604265	9	76905178	A	G	1.04 (1.03 to 1.05)	2.50E-19
28436984	rs35436838	9	77273910	G	T	1.07 (1.05 to 1.09)	1.27E-10
28436984	rs2378100	9	80513323	C	T	1.02 (1.02 to 1.03)	6.35E-10
28436984	rs4877387	9	81679875	T	C	1.02 (1.02 to 1.03)	2.20E-08
28436984	rs11534296	9	83282402	G	A	1.04 (1.03 to 1.05)	1.01E-17
28436984	rs7853970	9	86715566	T	C	1.05 (1.04 to 1.05)	2.19E-30
28436984	rs13283567	9	86764996	C	T	1.05 (1.03 to 1.06)	1.31E-15
28436984	rs1571536	9	92215638	T	C	1.03 (1.03 to 1.04)	2.19E-18
28436984	rs9330454	9	92515514	G	A	1.03 (1.02 to 1.04)	1.44E-13
28436984	rs10992769	9	96276910	C	G	1.03 (1.02 to 1.04)	1.11E-11
25231870	rs10816359	9	108757670	T	G	1.04 (1.02 to 1.06)	2.00E-08
28436984	rs10156597	9	108941509	A	T	1.11 (1.10 to 1.12)	5.04E-139
28436984	rs56927240	9	109148074	C	T	1.06 (1.05 to 1.07)	8.50E-28
28436984	rs10978641	9	109554196	T	A	1.03 (1.02 to 1.04)	5.10E-12
28436984	rs11792861	9	111809295	A	C	1.03 (1.02 to 1.04)	2.51E-14
25231870	rs10980854	9	114050357	A	G	1.06 (1.04 to 1.09)	1.00E-08
28436984	rs7852169	9	114318394	G	C	1.1 (1.09 to 1.12)	1.82E-46

28436984	rs2780243	9	120730928	C	T	1.02 (1.02 to 1.03)	2.31E-09
28436984	rs4836984	9	127405632	T	C	1.03 (1.03 to 1.04)	3.92E-19
28436984	rs467379	9	136905474	T	C	1.02 (1.02 to 1.03)	1.35E-08
28436984	rs7907759	10	1730008	A	G	1.04 (1.03 to 1.05)	2.43E-25
28436984	rs7912468	10	2697434	C	T	1.02 (1.02 to 1.03)	1.20E-09
28436984	rs1885740	10	10251910	G	A	1.03 (1.02 to 1.04)	3.35E-08
28436984	rs10906395	10	13541008	C	T	1.02 (1.02 to 1.03)	2.28E-09
28436984	rs61846901	10	51056858	C	T	1.03 (1.02 to 1.03)	1.21E-09
28436984	rs6415872	10	63660689	A	G	1.02 (1.02 to 1.03)	1.52E-09
28436984	rs5785580	10	65290254	A	AT	1.03 (1.02 to 1.04)	2.41E-12
28436984	rs7072571	10	71380093	A	G	1.03 (1.02 to 1.04)	2.69E-08
28436984	rs4746113	10	74071178	G	A	1.02 (1.02 to 1.03)	8.79E-09
28436984	rs77532868	10	88081438	T	C	1.06 (1.04 to 1.08)	3.31E-09
28436984	rs10617761	10	90266771	C	CAG	1.03 (1.02 to 1.04)	2.21E-08
28436984	rs1172955	10	97877320	T	A	1.04 (1.04 to 1.05)	6.06E-25
28436984	rs72842141	10	102686073	T	A	1.06 (1.05 to 1.08)	6.12E-12
28436984	rs59543819	10	103754188	C	T	1.03 (1.02 to 1.03)	1.97E-09
28436984	rs2066323	10	104871361	G	A	1.02 (1.02 to 1.03)	1.31E-09
28436984	rs10885077	10	112759731	T	G	1.02 (1.02 to 1.03)	2.95E-08
28436984	rs11349289	10	117483568	C	CA	1.03 (1.02 to 1.04)	2.28E-11
28436984	rs4751614	10	118696266	A	T	1.03 (1.02 to 1.04)	6.86E-11
28436984	rs10400136	10	120833948	G	A	1.03 (1.02 to 1.03)	2.73E-11
28436984	rs73435048	10	121154531	G	A	1.05 (1.03 to 1.06)	4.94E-08
25231870	rs12571664	10	121708929	T	C	1.04 (1.03 to 1.05)	3.00E-10
28436984	rs7077302	10	123676662	C	G	1.05 (1.04 to 1.07)	1.08E-12
28436984	rs9422857	10	126861278	G	C	1.03 (1.02 to 1.04)	3.67E-13
28436984	rs4576738	10	134294398	A	G	1.03 (1.02 to 1.04)	7.20E-10
28436984	rs3782120	11	206089	A	G	1.03 (1.03 to 1.04)	3.69E-14
28436984	rs10832021	11	13324530	G	A	1.05 (1.04 to 1.06)	6.27E-29
28436984	rs4359170	11	16596152	A	T	1.03 (1.02 to 1.04)	5.99E-12
28436984	rs1032682	11	22791324	T	C	1.02 (1.01 to 1.03)	2.69E-08
28436984	rs11606190	11	28033473	A	G	1.04 (1.03 to 1.05)	1.61E-13
28436984	rs6484408	11	28899164	G	A	1.03 (1.02 to 1.03)	6.63E-09
28436984	rs11031040	11	30317733	G	T	1.04 (1.03 to 1.05)	5.10E-15
28436984	rs1023955	11	43608835	G	T	1.03 (1.02 to 1.04)	7.36E-13
28436984	rs970179	11	45433845	A	G	1.02 (1.01 to 1.03)	3.53E-08
28436984	rs953230	11	46064974	A	G	1.03 (1.03 to 1.04)	5.52E-15
28436984	rs68002803	11	46539110	T	C	1.03 (1.02 to 1.04)	1.41E-10
28436984	rs59265730	11	47646640	A	AAAAAAC	1.03 (1.02 to 1.03)	7.30E-10
28436984	rs10897450	11	63593219	C	G	1.02 (1.02 to 1.03)	2.18E-09
28436984	rs10750766	11	65473798	C	A	1.03 (1.02 to 1.04)	9.96E-11
28436984	rs7115444	11	77555824	T	C	1.03 (1.02 to 1.04)	3.82E-12
28436984	rs4945266	11	78027488	G	A	1.05 (1.03 to 1.06)	1.17E-17
28436984	rs4402316	11	84780098	C	G	1.03 (1.02 to 1.04)	3.22E-11

28436984	rs7108556	11	86716236	T	C	1.03 (1.02 to 1.04)	2.62E-11
28436984	rs113557523	11	94085099	C	T	1.05 (1.03 to 1.06)	3.75E-11
28436984	rs6590889	11	101438191	C	T	1.04 (1.04 to 1.05)	8.62E-28
28436984	rs202185443	11	113949832	A	AAAAG	1.03 (1.02 to 1.04)	2.79E-08
28436984	rs17564430	11	115043574	G	T	1.04 (1.03 to 1.04)	2.07E-15
28436984	rs1815811	11	119059404	G	A	1.03 (1.02 to 1.04)	1.65E-12
28436984	rs7114175	11	122813983	T	A	1.06 (1.05 to 1.07)	1.14E-56
28436984	rs77530428	12	17126283	G	A	1.13 (1.10 to 1.17)	6.25E-14
28436984	rs10842343	12	24579079	T	A	1.03 (1.02 to 1.03)	5.78E-11
28436984	rs7971408	12	47876942	T	C	1.05 (1.03 to 1.06)	9.36E-13
28436984	rs1054442	12	49389320	A	C	1.04 (1.03 to 1.04)	7.00E-19
28436984	rs1131017	12	56435929	C	G	1.02 (1.02 to 1.03)	3.75E-09
28436984	rs1148006	12	75978358	G	A	1.03 (1.02 to 1.04)	5.81E-09
28436984	rs9943694	12	84222209	C	A	1.03 (1.02 to 1.04)	1.41E-08
28436984	rs7979001	12	97506357	A	G	1.02 (1.01 to 1.03)	6.13E-09
28436984	rs3764002	12	108618630	C	T	1.03 (1.02 to 1.04)	1.21E-11
28436984	rs11065822	12	111600134	T	G	1.03 (1.02 to 1.03)	1.96E-09
28436984	rs474463	12	115107376	C	T	1.03 (1.02 to 1.04)	1.65E-09
28436984	rs7133066	12	117360617	T	G	1.03 (1.02 to 1.05)	4.21E-09
28436984	rs660549	12	121300988	C	T	1.02 (1.01 to 1.03)	3.36E-08
28436984	rs5801963	12	132412187	A	AG	1.03 (1.02 to 1.04)	2.94E-08
28436984	rs9548873	13	40238492	C	T	1.03 (1.02 to 1.04)	7.50E-15
28436984	rs73187215	13	42646769	G	A	1.04 (1.03 to 1.05)	1.34E-08
28436984	rs9568123	13	49475780	G	A	1.03 (1.02 to 1.04)	4.06E-08
28436984	rs4886140	13	59833519	A	G	1.03 (1.02 to 1.04)	3.10E-12
28436984	rs1925047	13	74600274	C	A	1.03 (1.03 to 1.04)	5.70E-17
25231870	rs1324913	13	74635588	G	T	1.03 (1.02 to 1.04)	3.00E-10
28436984	rs11619721	13	112082513	G	T	1.04 (1.03 to 1.06)	1.33E-08
25231870	rs9560113	13	112183348	G	A	1.05 (1.04 to 1.06)	2.00E-17
28436984	rs9522262	13	112186283	C	G	1.04 (1.03 to 1.05)	1.69E-25
28436984	rs74499585	13	112285043	A	G	1.06 (1.04 to 1.08)	1.55E-13
28436984	rs10136330	14	30514335	C	T	1.06 (1.04 to 1.08)	6.65E-09
28436984	rs35035068	14	40968669	A	AT	1.03 (1.02 to 1.03)	2.49E-10
28436984	rs10138913	14	60943106	T	C	1.06 (1.05 to 1.07)	1.14E-41
25231870	rs1958560	14	66036795	A	G	1.03 (1.02 to 1.04)	4.00E-08
28436984	rs10143972	14	93850179	C	T	1.04 (1.03 to 1.05)	3.05E-15
28436984	rs10145469	14	97769834	C	A	1.06 (1.04 to 1.08)	5.56E-11
28436984	rs941520	14	99709702	C	A	1.02 (1.01 to 1.03)	8.99E-09
28436984	rs12894936	14	100846991	C	T	1.05 (1.04 to 1.06)	3.73E-34
25231870	rs7141210	14	101182470	T	C	1.03 (1.02 to 1.04)	6.00E-09
28436984	rs6575806	14	101353211	C	A	1.03 (1.02 to 1.05)	9.05E-09
28436984	rs79084266	14	101367407	G	C	1.04 (1.03 to 1.06)	4.27E-08
28436984	rs7178532	15	23794517	A	G	1.04 (1.04 to 1.05)	2.29E-26
28436984	rs184950120	15	23810843	C	T	1.49 (1.33 to 1.66)	9.78E-13

25231870	rs12148769	15	24152094	G	A	1.05 (1.03 to 1.07)	5.00E-11
28436984	rs8040272	15	24824016	A	G	1.05 (1.03 to 1.06)	7.79E-13
28436984	rs34513772	15	40608820	T	C	1.02 (1.02 to 1.03)	6.20E-09
28436984	rs4924538	15	41494364	T	A	1.03 (1.02 to 1.03)	1.03E-09
28436984	rs1435753	15	47925066	C	T	1.03 (1.02 to 1.04)	2.91E-12
28436984	rs28757192	15	51507610	C	T	1.06 (1.04 to 1.09)	3.50E-09
28436984	rs11852771	15	54364552	A	G	1.02 (1.02 to 1.03)	9.69E-10
28436984	rs72756954	15	64537300	C	G	1.06 (1.04 to 1.08)	8.45E-13
28436984	rs10153031	15	67987293	T	G	1.04 (1.03 to 1.05)	6.05E-25
28436984	rs35510813	15	77810351	T	TA	1.03 (1.02 to 1.04)	2.96E-09
28436984	rs1971554	15	83406228	T	C	1.03 (1.02 to 1.04)	2.35E-13
28436984	rs12915845	15	89042467	C	T	1.04 (1.03 to 1.05)	3.70E-25
28436984	rs148825694	15	99299706	T	TATC	1.06 (1.04 to 1.08)	6.61E-09
28436984	rs1704528	16	14388750	C	T	1.05 (1.04 to 1.06)	1.50E-35
28436984	rs153793	16	15542199	G	A	1.02 (1.02 to 1.03)	9.27E-10
28436984	rs112991346	16	19967668	C	T	1.05 (1.03 to 1.06)	6.47E-16
28436984	rs4780885	16	20380004	C	G	1.03 (1.02 to 1.03)	1.52E-11
28436984	rs113388806	16	24804954	T	A	1.06 (1.04 to 1.09)	1.26E-09
28436984	rs8051833	16	29896390	G	A	1.04 (1.03 to 1.05)	9.26E-22
25231870	rs1129700	16	29918034	T	C	1.03 (1.02 to 1.04)	2.00E-09
28436984	rs3809624	16	30102802	C	T	1.03 (1.02 to 1.04)	3.64E-12
28436984	rs143461173	16	52283158	A	G	1.03 (1.02 to 1.04)	6.10E-09
28436984	rs9972653	16	53814363	G	T	1.05 (1.04 to 1.06)	6.47E-40
28436984	rs7359336	16	69733460	G	A	1.05 (1.05 to 1.06)	5.33E-44
28436984	rs4448948	16	72569236	T	A	1.04 (1.03 to 1.06)	4.33E-08
28436984	rs112752732	17	1942577	C	G	1.06 (1.04 to 1.08)	7.26E-09
28436984	rs142643995	17	2017993	T	C	1.07 (1.04 to 1.09)	3.25E-08
25231870	rs7215990	17	6034831	G	A	1.04 (1.03 to 1.05)	2.00E-08
28436984	rs55680968	17	7774047	G	A	1.05 (1.03 to 1.06)	1.17E-09
28436984	rs59246405	17	43123625	T	C	1.03 (1.02 to 1.04)	2.03E-12
28436984	rs11079810	17	46227846	T	C	1.04 (1.02 to 1.05)	9.62E-09
28436984	rs9635759	17	49613785	A	G	1.06 (1.05 to 1.07)	2.78E-46
28436984	rs2787487	17	53209382	C	G	1.03 (1.02 to 1.04)	1.62E-15
28436984	rs7218751	17	77796437	A	G	1.03 (1.02 to 1.04)	1.37E-11
28436984	rs59652033	17	77951023	C	T	1.03 (1.02 to 1.04)	1.16E-09
28436984	rs66508321	17	78739672	G	A	1.03 (1.02 to 1.04)	2.84E-13
28436984	rs2659007	17	79217478	G	A	1.03 (1.02 to 1.04)	6.23E-15
28436984	rs12937034	17	79446015	G	A	1.03 (1.02 to 1.03)	6.43E-10
28436984	rs11873906	18	3813464	G	A	1.05 (1.04 to 1.06)	2.33E-32
28436984	rs8087304	18	31765736	A	T	1.02 (1.01 to 1.03)	6.54E-09
28436984	rs1512238	18	44748467	G	A	1.06 (1.05 to 1.06)	2.48E-44
25231870	rs2137289	18	44752125	A	G	1.05 (1.04 to 1.06)	8.00E-20
28436984	rs7239114	18	45921214	G	A	1.02 (1.01 to 1.03)	4.02E-08
28436984	rs3746037	19	1828948	A	C	1.04 (1.03 to 1.05)	1.01E-13

28436984	rs169080	19	4980864	C	T	1.03 (1.02 to 1.03)	1.33E-10
28436984	rs484353	19	7891767	A	G	1.03 (1.02 to 1.04)	8.28E-16
28436984	rs4804117	19	9984509	T	G	1.05 (1.04 to 1.05)	3.58E-31
25231870	rs889122	19	9995867	G	T	1.04 (1.03 to 1.05)	2.00E-13
28436984	rs10422323	19	13104027	A	G	1.04 (1.03 to 1.05)	2.01E-09
28436984	rs12460047	19	18346228	G	A	1.03 (1.02 to 1.04)	6.93E-11
28436984	rs11668587	19	18829770	G	A	1.03 (1.03 to 1.04)	1.86E-15
28436984	rs56367141	19	31051857	C	A	1.04 (1.03 to 1.05)	2.11E-11
28436984	rs77563037	19	36208693	C	CG	1.03 (1.03 to 1.04)	1.92E-16
28436984	rs4804025	19	47609223	G	A	1.04 (1.03 to 1.05)	3.11E-22
28436984	rs2548458	19	49209325	T	C	1.02 (1.01 to 1.03)	3.53E-08
28436984	rs4801809	19	50334895	C	T	1.04 (1.03 to 1.06)	1.33E-09
28436984	rs2889128	19	58973929	A	C	1.02 (1.01 to 1.03)	7.57E-09
25231870	rs852069	20	17122593	G	A	1.04 (1.03 to 1.05)	1.00E-13
28436984	rs1535252	20	19682834	C	T	1.03 (1.02 to 1.03)	4.82E-11
28436984	rs111558392	20	20348962	C	T	1.04 (1.03 to 1.05)	2.09E-13
28436984	rs4813429	20	21485806	T	C	1.03 (1.02 to 1.04)	4.17E-09
28436984	rs1737894	20	31054702	C	G	1.02 (1.01 to 1.03)	1.91E-08
28436984	rs2295094	20	33447915	A	G	1.04 (1.03 to 1.05)	5.86E-12
28436984	rs36093651	20	37287102	T	C	1.04 (1.03 to 1.05)	6.51E-16
28436984	rs2425674	20	43529461	G	C	1.02 (1.01 to 1.03)	2.35E-08
28436984	rs3746619	20	54823805	A	C	1.05 (1.03 to 1.06)	5.52E-12
28436984	rs13043968	20	54830983	C	A	1.04 (1.03 to 1.05)	1.92E-11
28436984	rs443252	20	62799680	T	C	1.06 (1.04 to 1.08)	1.29E-10
28436984	rs62229372	21	37692507	T	C	1.05 (1.04 to 1.06)	3.51E-17
28436984	rs117143374	21	40555561	C	T	1.05 (1.04 to 1.06)	7.98E-19
25231870	rs2836950	21	40604429	C	G	1.03 (1.02 to 1.04)	6.00E-11
28436984	rs151680	22	22273242	T	C	1.03 (1.02 to 1.04)	2.52E-13
28436984	rs5753377	22	31293700	G	A	1.03 (1.02 to 1.04)	8.04E-13
28436984	rs4303811	22	39157755	G	A	1.04 (1.02 to 1.05)	3.19E-09
28436984	rs9614460	22	45745229	G	T	1.03 (1.02 to 1.03)	1.38E-09
28436984	rs8136272	22	49678782	A	T	1.04 (1.03 to 1.05)	6.18E-20
28436984	rs55840812	23	30348613	C	CT	1.04 (1.03 to 1.05)	1.26E-12
28436984	rs35334917	23	50150036	C	CAT	1.03 (1.02 to 1.05)	8.76E-11
28436984	rs2885287	23	68377499	A	G	1.04 (1.02 to 1.05)	4.27E-10
28436984	rs55710677	23	71411234	T	C	1.03 (1.02 to 1.05)	4.25E-08
28436984	rs5920843	23	99955037	A	G	1.04 (1.03 to 1.05)	1.16E-10
28436984	rs6621284	23	101250436	A	G	1.03 (1.02 to 1.04)	2.35E-08
28436984	rs36068064	23	109292803	T	C	1.03 (1.02 to 1.04)	8.15E-10
28436984	rs1989318	23	130502089	G	A	1.05 (1.04 to 1.06)	1.32E-18
28436984	rs148395841	23	135971387	G	GT	1.04 (1.03 to 1.05)	4.82E-17

† means that the chromosome physical position is based on the National Center for Biotechnology Information database, Build 37; PMID = PubMed Unique Identifier; SNP = single nucleotide polymorphism; Chr = chromosome; OR = odds ratio; CI =

confidence interval.

Supplementary Table 3. Characteristics of the selected studies in meta-analyses

First author, publication year, Country, Study design	Cases/non-cases, age, duration of follow up	OR or RR (95% CI) of ovarian cancer	OR or RR (95% CI) of serous ovarian cancer	Adjusted factors	Quality scores† (selection of study groups, comparability of groups*, ascertainment of the exposure or outcome§)
Fujita M, 2008, Japan, Hospital-based case-control study	130/1837, 57.2 (mean)	0.81 (0.69 to 0.94)		Age, year of survey, referral base, area of residence, smoking history, history of alcohol drinking, family history of index cancer in patients and siblings, occupation and parity number.	8 (3, 2, 3)
Tavani A, 1993, Italy, Hospital-based case-control study	193/709, <45	0.84 (0.73 to 0.97)		Age, education, family history, number of births, number of abortions and oral contraceptive use.	7 (2, 2, 3)
Shu XO, 1989, China, Population-based case-control study	172/172, 49 (mean)	0.84 (0.71 to 0.99)		Age, education, number of live births, ovarian cyst.	7 (3, 2, 2)
Yang HP, 2016, Poland, Case-control study	302/1356, 20-74 (range)	0.87 (0.78 to 0.98)		Age (in 5-year age categories) and study site (Lodz or Warsaw), age at menopause (<45, 45–49, 50–54, or ≥55 years), oral contraceptive use (never, ever), and number of live births (0, 1, 2, or ≥3)	8 (4, 2, 2)
Wu ML, 1988, United States, Case-control study	297/1000, 18-85 (range)	0.91 (0.84 to 0.99)		Age	6 (3, 1, 2)

Greggi S, 2000, Italy, Hospital-based case-control study	426/868, 13-80 (range)	0.95 (0.86 to 1.04)		Age, education, parity, oral contraceptive use, family history of ovarian cancer, menopausal status, and age at menopause.	7 (2, 2, 3)
Merritt MA, 2013, USA, Population-based case-control study	1571/2100, 52.5 (mean)	0.95 (0.89 to 1.03)		Age (continuous), study center (Massachusetts, New Hampshire, The United States of America), study phase (1992–1997, 1998–2003, 2003–2008), parity (0, 1, 2, >2), OC pill use (0, 3 months to <5 years, ≥5 years), family history of ovarian cancer (yes/no), family history of breast cancer (yes/no) and tubal ligation (yes/no)	7 (3, 2, 2)
Hankinson SE, 1995, United States, Cohort study	258/121442, 30-55 (range), 12	0.96 (0.88 to 1.05)		Age, parity, duration of oral contraceptive use, tuba1 ligation, age at menopause, smoking status, Quetelet's Index.	7 (2, 2, 3)
Booth M, 1989, United Kingdom, Hospital-based case-control study	232/448, 51.9 (mean)	0.96 (0.85 to 1.08)		Age and social class.	6 (2, 1, 3)
Chiaffarino F, 2001, Italy, Hospital-based case-control study	1028/2403, <=79	0.96 (0.91 to 1.02)		Age, center, education, parity, oral contraceptive use, and family history of ovarian and breast cancer in first degree relatives.	7 (2, 2, 3)

Riman T, 2002, Sweden, Population-based case-control study	584/3555, 62.9 (mean)	0.97 (0.91 to 1.03)		Age, parity, body mass index, age at menopause, and duration of oral contraceptive use as categorized variables and for ever use of hormone replacement therapy.	6 (3, 2, 1)
Shin A, 2011, Korea, Cohort study	514/443395, 30-80 (range), 12	0.97 (0.91 to 1.03)		Age, body mass index, income level, alcohol consumption, cigarette smoking, menopausal status, age at the first live birth and breastfeeding.	7 (2, 2, 3)
Weiderpass E, 2012, Japan, Cohort study	86/45662, 40-69 (range, at enrollment), 7.6	0.97 (0.85 to 1.11)		Nulliparous, parity, age at first birth, breastfeeding, use of exogenous hormones, menopausal status at enrollment, height, body mass index, smoking status, exposure to second-hand smoke, physical activity, usual sleep duration, family history of cancer in first-degree relative.	6 (3, 1, 2)
Gay GMW, 2015, Singapor, Cohort study	107/28094 (48 serous), 57 (mean, at enrollment), 17	0.98 (0.86 to 1.12)	0.96 (0.79 to 1.16)	Age (continuous), housing type (1–3 room flat, C4 room flat, private or landed property, others) and family history of breast cancer	9 (4, 2, 3)
Yang HP, 2012, USA, Cohort study	849/168542 (451 serous), 62.3 (mean, at enrollment), 7.5 (mean)	0.99 (0.94 to 1.04)	0.97 (0.90 to 1.04)	Age (continuous), oral contraceptive use (ever/never), parity (yes/no), menopausal hormone therapy (ever/never)	8 (3, 2, 3)

Fortner RT, 2015, Europe, Cohort study	1245/332881 (611 serous), 25-75 (range), 18	1.00 (0.96 to 1.05)	1.00 (0.95 to 1.06)	Age, ever full-term pregnancy, ever oral contraceptive use, menopausal status at recruitment and age at menopause	8 (3, 2, 3)
Polychronopoulou A, 1993, Greece, Hospital-based case-control study	189/200, <=75	1.01 (0.80 to 1.28)		Age, years of schooling, weight before the onset of the present disease, menopausal status, parity, tobacco smoking, average consumption of alcoholic beverages and average coffee drinking	8 (3, 2, 3)
Riman T, 2001, Sweden, Population-based case-control study	169/3555, 50-74 (range)	1.02 (0.92 to 1.13)		Age, parity, body mass index, and age at menopause as categorized variables and ever use of oral contraceptive, unopposed estrogens with cyclic progestins, and estrogens with continuous progestins.	7 (4, 2, 1)
Parazzini F, 1991, Italy, Hospital-based case-control study	91/273, <65	1.02 (0.85 to 1.22)		Age, education, parity, oral contraceptive use, age at menopause, menopausal status, and lifelong menstrual cycle pattern.	7 (2, 2, 3)
Le ND, 2014, Canada, Population-based case-control study	608/335, 20–79 (range)	1.02 (0.89 to 1.16)		Age.	6 (4, 1, 1)
Purdie D, 1995, Australia, Population-based case-control study	822/859, 18-79 (range)	1.03 (0.96 to 1.11)		Age, parity.	8 (4, 2, 2)

Kurta ML, 2012, USA, Population-based case-control study	902/1802, >=25	1.06 (0.98 to 1.14)		Age (continuous), race (white, black, other), and education (non-high school graduate, high school graduate, post high-school)	6 (3, 1, 2)
Le DC, 2012, Northern Vietnam, Case-control study	262/755, 48 (median)	1.09 (0.96 to 1.22)		Age, education level, parity, body mass index, menopausal status and oral contraceptive use	9 (4, 2, 3)
Salazar-Martinez E, 1999, Mexico, Hospital-based case-control study	84/668, 53.7 (mean)	1.11 (0.98 to 1.27)		Age, anovulatory index, smoking, diabetes mellitus, hypertension, physical activity, menopausal status, and body mass index.	6 (2, 2, 2)
Jordan SJ, 2005, Australia, Population-based case-control study	403/829 (serous), 54.5 (mean)		1.01 (0.91 to 1.13)	Age, weight at age 20, parity, ever use of oral contraceptive, and height.	8 (4, 2, 2)
Our study, 2018, China, Case-control study	289/206 (143 serous), 55.3 (mean)	0.90 (0.82 to 0.99)	0.92 (0.82 to 1.04)	Age at diagnosis, body mass index (continuous), family history of cancer.	7 (4, 2, 1)

[†]Quality scores were based on Newcastle-Ottawa Scale (http://www.ohri.ca/programs/clinical_epidemiology/oxford.asp); [*] Studies that controlled for age received one star, whereas studies that controlled for other ovarian cancer-related covariates (e.g., parity, use of oral contraceptive, family history of ovarian cancer, body mass index) received an additional star; [§]A case-control study with a response rate >= 90% was assigned one star, and a cohort study with a follow-up time >= 7 years was assigned one star, the same as a cohort study with a follow-up rate >= 90%; OR = odds ratio; RR = relative risk; CI = confidence interval.

Supplementary Table 4. Association of the weighted genetic score with confounders

	EOC		SOC	
**Variable**	**OR (95% CI)**	***P***	**OR (95% CI)**	***P***
Age	0.95 (0.87 to 1.03)	0.217	0.99 (0.89 to 1.10)	0.891
Body mass index (kg/m^2)	0.97 (0.88 to 1.05)	0.432	0.98 (0.88 to 1.09)	0.687
Family history of cancer	0.98 (0.90 to 1.08)	0.725	1.00 (0.90 to 1.11)	0.977

EOC = epithelial ovarian cancer; SOC = serous ovarian cancer; OR = odds ratio; CI = confidence interval.

Supplementary Figure 1. Further studies in meta-analyses

(a) Sensitivity analyses in case-control studies

Exclude	OR (95% CI)	*P*
None	0.970 (0.938 to 1.003)	0.071
Shu XO, 1989, China	0.974 (0.943 to 1.007)	0.118
Tavani A, 1993, Italy	0.976 (0.945 to 1.007)	0.133
Our study, 2018, China	0.975 (0.943 to 1.009)	0.149
Wu ML, 1988, United Stated	0.975 (0.942 to 1.009)	0.152
Greggi S, 2000, Italy	0.971 (0.937 to 1.006)	0.108
Merritt MA, 2013, USA	0.971 (0.936 to 1.007)	0.114
Booth M, 1989, United Kingdom	0.970 (0.937 to 1.005)	0.094
Chiaffarino F, 2001, Italy	0.970 (0.934 to 1.007)	0.111
Riman T, 2002, Sweden	0.969 (0.934 to 1.006)	0.104
Polychronopoulou A, 1993, Greece	0.969 (0.936 to 1.003)	0.071
Riman T, 2001, Sweden	0.967 (0.933 to 1.001)	0.057
Le ND, 2014, Canada	0.967 (0.934 to 1.002)	0.061
Purdie D, 1995, Australia	0.964 (0.931 to 0.998)	0.036
Kurta ML, 2012, USA	0.962 (0.931 to 0.994)	0.019
Salazar-Martinez E, 1999, Mexico	0.964 (0.934 to 0.995)	0.024

Exclude, the study excluded in the sensitivity analysis; OR = odds ratio; CI = confidence interval.

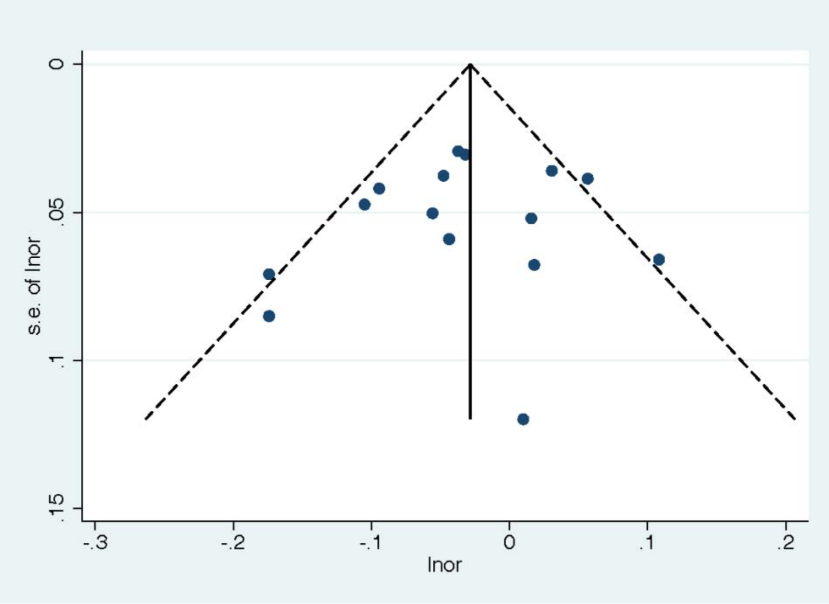

(b) Funnel plot with pseudo 95% confidence limits for case-control studies. lnor = ln(odds ratio); s.e. = standard error.

(c) Sensitivity analyses in cohort studies

Exclude	RR (95% CI)	*P*
None	0.992 (0.963 to 1.021)	0.574
Weiderpass E, 2012, Japan	0.993 (0.963 to 1.023)	0.633
Gay GMW, 2015, Singapor	0.992 (0.963 to 1.023)	0.613
Fortner RT, 2015, Europe	0.981 (0.943 to 1.021)	0.347
Hankinson SE, 1995, United Stated	0.996 (0.966 to 1.027)	0.798
Yang HP, 2012, USA	0.992 (0.957 to 1.028)	0.659

Exclude, the study excluded in the sensitivity analysis; RR = relative risk; CI = confidence interval.

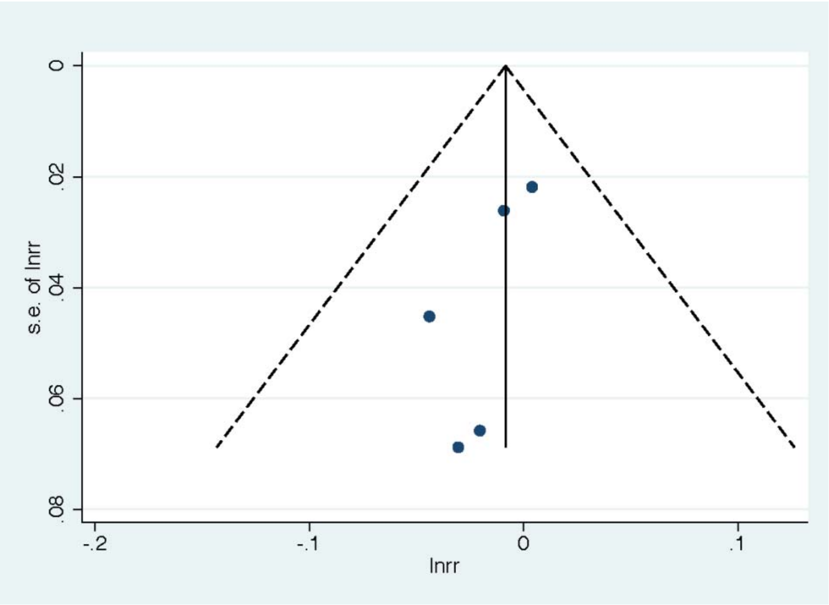

(d) Funnel plot with pseudo 95% confidence limits for cohort studies. lnrr = ln(relative risk); s.e. = standard error.

(e) Meta-analyses of associations between age at menarche and ovarian cancer risk

	Case-control			Cohort		
	N	OR (95% CI)	*P*	N	RR (95% CI)	*P*
All	19	0.964 (0.932 to 0.998)	0.036	6	0.988 (0.962 to 1.014)	0.366
Asian	4	0.908 (0.800 to 1.032)	0.139	3	0.971 (0.921 to 1.024)	0.279
European	9	0.955 (0.925 to 0.986)	0.005	1	1.004 (0.962 to 1.048)	0.855
Other	6	1.005 (0.950 to 1.064)	0.857	2	0.982 (0.940 to 1.027)	0.430

N, the number of studies analyzed; OR = odds ratio; RR = relative risk; All, all populations included; Asian, only Asian population included; European, only European population included; Other, Asian or European population excluded.

(f) Meta-analyses of associations between age at menarche and serous ovarian cancer risk

	Case-control			Cohort		
	N	OR (95% CI)	*P*	N	RR (95% CI)	*P*
All	2	0.970 (0.896 to 1.050)	0.450	3	0.986 (0.945 to 1.029)	0.514

N, the number of studies analyzed; OR = odds ratio; RR = relative risk; All, all populations included.
